# Childhood adversity and parent perceptions of child resilience

**DOI:** 10.1186/s12887-018-1170-3

**Published:** 2018-06-26

**Authors:** Nia Heard-Garris, Matthew M. Davis, Moira Szilagyi, Kristin Kan

**Affiliations:** 1 Robert Wood Johnson Foundation Clinical Scholars Program, Ann Arbor, MI USA; 2 Department of Pediatrics and Communicable Diseases, University of Michigan, 2800 Plymouth Rd. Bldg. 14, Room G100, Ann Arbor, MI 48109-2800 USA; 3 Division of Academic General Pediatrics, Department of Pediatrics, Ann & Robert H. Lurie Children’s Hospital of Chicago and Northwestern University Feinberg School of Medicine, Chicago, IL USA; 4 Mary Ann & J. Milburn Smith Child Health Research, Outreach, and Advocacy Center, Stanley Manne Children’s Research Institute, Ann & Robert H. Lurie Children’s Hospital of Chicago, 225 East Chicago Ave, Box 162, Chicago, IL 60611 USA; 5 Departments of Medical Social Sciences, Medicine, and Preventive Medicine, Northwestern University Feinberg School of Medicine, 225 East Chicago Ave, Box 162, Chicago, IL 60611 USA; 6 Mattel Children’s Hospital, Department of Pediatrics Developmental Studies Program, David Geffen School of Medicine and University of California Los Angeles 200 UCLA Medical Plaza Suite 265, California, Los Angeles 90095 USA; 7 Present Address: 225 East Chicago Ave, Box 162, Chicago, IL 60611 USA

**Keywords:** Adverse childhood experiences, ACEs, Resilience, Primary care

## Abstract

**Background:**

Adverse childhood experiences (ACEs) negatively impact health throughout the life course. For children exposed to ACEs, resilience may be particularly important. However, the literature regarding resilience, particularly the self-regulation aspect of resilience, is not often described in children with ACEs. Additionally, family and community factors that might help promote resilience in childhood may be further elucidated. We aimed to describe the relationship between ACEs and parent-perceived resilience in children and examine the child, family, and community-level factors associated with child resilience.

**Methods:**

Using the US-based, 2011–2012 National Survey of Children’s Health, we examined adverse childhood experiences (NSCH-ACEs) as the main exposure. Affirmative answers to adverse experiences generated a total parent-reported NSCH-ACE score. Bivariate and multivariable logistic regression models were constructed for parent-perceived child resilience and its association with ACEs, controlling for child, family, and neighborhood-level factors.

**Results:**

Among 62,200 US children 6–17 years old, 47% had 0 ACEs, 26% had 1 ACE, 19% had 2–3 ACEs, and 8% had 4 or more ACEs. Child resilience was associated with ACEs in a dose-dependent relationship: as ACEs increased, the probability of resilience decreased. This relationship persisted after controlling for child, family, and community factors. Specific community factors, such as neighborhood safety (*p* < .001), neighborhood amenities (e.g., libraries, parks) (*p* < .01) and mentorship (*p* < .05), were associated with significantly higher adjusted probabilities of resilience, when compared to peers without these specific community factors.

**Conclusions:**

While ACEs are common and may be difficult to prevent, there may be opportunities for health care providers, child welfare professionals, and policymakers to strengthen children and families by supporting community-based activities, programs, and policies that promote resilience in vulnerable children and communities in which they live.

**Electronic supplementary material:**

The online version of this article (10.1186/s12887-018-1170-3) contains supplementary material, which is available to authorized users.

## Background

Children exposed to adverse childhood experiences, or ACEs, experience biological and social disadvantages throughout the life course. However, the capacity for this population to demonstrate resilience, − that is, the ability to withstand difficulties—in childhood remains unclear. Originally, ACEs were described as ten experiences that were categorized into 3 major experiences: abuse, neglect, and intra-familial stressors that contribute to household dysfunction (i.e., witnessing domestic violence; and household members with mental illness, substance abuse, or incarceration histories) [1]. The initial set of ACEs [1] have been expanded to include other types of experiences, such as community violence and racial discrimination, among other experiences. The original and expanded ACEs have been a major focus of study due to the strong associations of ACEs with negative health behaviors [2, 3] and marked outcomes over the life course [4–6]. For example, individuals exposed to ACEs are more likely to have ischemic heart disease, diabetes, cancer, alcoholism, and use illicit drugs [7]. ACE exposure has also been correlated with below-average literacy and language skills, which may in turn, limit a child’s academic potential [8, 9]. Mechanistically, ACEs are thought to alter gene expression that may induce changes to the developing brain, including chronic inflammation and retarded neuronal growth and survival, giving rise to structural changes that persist into adulthood [10–12]. Such modifications in brain architecture [12] and subsequent genetic insults [10] may substantively determine a child’s trajectory after experiencing hardship, especially in the absence of protective factors [12, 13].

While some ACE-exposed children experience biopsychosocial challenges, others do not. This may be due to the presence of protective factors that nurture an individual’s resilience and mitigate the consequences of ACEs. Resilience, or the ability to rebound from significant challenges, may impart a buffering effect on the development of negative outcomes into adulthood [14]. Currently, there is no consensus regarding the definition and operationalization of resilience. Resilience may be conceptualized as either a static trait or set of predictive traits, [15, 16] as a dynamic, evolving process or processes, or both [15–17]. Resilience may also be defined with respect to outcomes. Resilience may be viewed as the absence of negative outcomes or the presence of positive outcomes. Due to these differences, resilience has been studied from multiple perspectives [16, 18].

Resilience in children and young adults has been correlated with individual characteristics, such as problem-solving ability, self-efficacy, optimism, and autonomy [18, 19]. Resilience has also been associated with the presence of close relationships with others such as parents, friends, and romantic partners [14, 16, 20, 21].

While fundamentally, safe, stable, and nurturing relationships are considered the cornerstone of resilience in children, [16, 17, 19, 21, 22] the typical attachment of the caregiver-child relationship may make the development of resilience difficult for children with ACEs. Further, disruptions in the household may require children to more heavily depend on their own individual traits, in addition to family and community-based supports. For children with ACEs, those individual traits may be even more important to their overall trajectory.

More specifically, understanding self-regulation, an important aspect of resilience,[23, 24] may optimize a child’s development and health throughout the life course. Self-regulation is described as an individual’s ability to set goals, plan, and execute tasks, while adjusting or maintaining behavioral, emotional, or attentional stability [25]. Self-regulation in the context of stress, such as ACEs, may be regarded not only as a key factor or predictor of resilience, but in essence a source of resilience [23, 24, 26]. Artuch-Garde et al., found that that learning from mistakes, an important factor of self-regulation, is predictive of resilience. Further, an individual’s drive to identify solutions when faced with a challenge embodies a central component of resilience [26].

Though the conceptualization of resilience is complex, due to both the reliance on individual traits and skill development, it is well acknowledged that resilience is influenced and maintained by factors outside of the child. These external factors are framed by the Bronfenbrenner socio-ecological model, which proposes that child development is shaped by the immediate environment, such as caregiver relationships as well as the cultural and community environment [27]. Thus, these elements are important considerations when studying positive child development [27]. Children with ACEs may depend on their communities more heavily to help foster resilience, further necessitating the identification of specific resilience-promoting community factors. Although there has been some attention to community supports, such as the influence of schools and teachers on childhood resilience, [17] there has been less focus on other specific community factors, such as the presence of neighborhood assets, like libraries and parks, as levers for fostering resilience in children.

Taken together, both understanding the influence of ACEs on a child’s resilience and identifying family and community pro-resilience characteristics, may guide the development of interventions targeted at at-risk children and possibly buffer subsequent negative health outcomes [14]. However, much of the ACE literature is focused on adult cohorts reporting on ACEs retrospectively, which makes resilience in childhood difficult to ascertain. Therefore, in this paper, we aimed to examine: 1) the relationship between ACEs and parent-perceived resilience in children, using a US-based nationally representative cohort of children; and 2) to describe child, family, and community factors associated with resilience in children. We hypothesized that as children are exposed to more ACEs, parent-perceived resilience would be lower. We also hypothesized that children with more family and community supports would be have higher parent reports of resilience.

## Methods

### Data source

We use data from the 2011–2012, National Survey of Children’s Health (NSCH), conducted by the Centers for Disease Control and Prevention’s National Center for Health Statistics. The NSCH is a United-States-based, nationally representative, cross-sectional, telephone survey of households with children 0–17 years old. The National Center for Health Statistics, State and Local Area Integrated Telephone Survey program randomly sampled United States telephone numbers and interviewed the parent or guardian in the household most knowledgeable about the child’s health or health care use [28]. The 2011 NSCH dataset includes 95,677 children (overall response rate 38.2%), from all 50 states and the District of Columbia. Survey design and methodology are documented elsewhere [29, 30]. The NSCH dataset analyzed in this current study is available in the Data Resource Center for Child & Adolescent Health repository [http://childhealthdata.org/help/dataset] [30].

### Measures

#### Outcome measure

Parent-perceived resilience was ascertained with a question administered to parents of children 6–17 years old, “How often is this true: he/she stays calm and in control when faced with a challenge?” This question has been used previously to describe parent-perceived resilience within this dataset [29, 30] and was created and selected by a technical expert panel. Also, this conceptualization is aligned with the component of self-regulation that is predictive of resilience [26]. Parental answers of “never”, “rarely”, and “sometimes,” represented 32.6% of the sample and were collapsed so that those answers were considered not demonstrating resilience. Answers of “usually” and “always” represented 67.4% of the sample and were collapsed as demonstrating resilience [31].

#### Exposure measure

The primary independent variable was a composite score of nine adverse childhood experiences that were parent-reported in the National Survey of Children’s Health, called NSCH-ACEs. The experiences asked in the NSCH were: 1) material and financial hardship, 2) divorce of a parent, 3) death of a parent, 4) having a parent who is in jail or prison, 5) exposure to domestic violence, 6) exposure to violence in their neighborhood, 7) living with someone with mental illness, 8) exposure to drug or alcohol abuse, and 9) experiencing racism. Each experience was coded as a binary outcome of whether the child experienced the stressor or not, and a composite score of the ACEs was generated based on the total number of affirmative answers to ACEs for each child. This composite variable has been used previously and its coding is publicly available in the NSCH variable codebooks [31].

#### Covariates

Individual, family, and community level factors were used as covariates to examine the relationship between resilience and ACEs.

Child-level factors included: age; sex; race/ethnicity (non-Hispanic white, non-Hispanic black, Hispanic and other); and special health care needs status. Family actors included: household income-to-poverty ratio (< 100%, 101–133%, 134–200, > 200% of the federal poverty level [FPL]); highest education attained by parents (less than high school, high school graduate, or greater than high school); total number of children in the household; and family structure (2 parents, single mother, or other). Additional family factors such as eating a meal together, religious attendance, and sharing ideas with children were also included. Community factors included neighborhood cohesion, safety, amenities (i.e., presence of sidewalks, parks, recreation centers, or libraries), and detractors (i.e., litter, rundown housing, graffiti). A measure of mentorship (i.e., the presence of a non-relative adult mentor for the child) was also included. These co-variates were selected, drawing from Bronfenbrenner’s socio-ecological model, presuming that children with positive family and community supports would positively contribute to resilience regardless of ACE exposure. Additional file 1: Table S1 lists the questions that comprise the exposure and outcome variables, along with the covariates.

#### Analysis

The analysis sample was restricted to children 6–17 years old without missing data with respect to the resilience measure and the composite ACE variable (*n* = 62,200, 65% of the overall sample). For bivariate analyses of ACEs and covariates of interest, we modeled ACEs as a categorical variable (0 ACEs, 1 ACE, 2–3 ACEs, and 4 or more ACEs). For multivariable analyses, we used logistic regression to estimate the relative odds of resilience for each accumulated ACE (continuous variable) after adjusting for the child-level, family-level, and community-level factors listed above. In this model, we also included a quadratic term for the ACE variable, as we found that as ACEs accumulated, the association with resilience was a non-linear relationship (e.g., adding additional ACEs modified the relationship between ACEs and resilience). Adjusted probability estimates of resilience were calculated and adjusted after holding covariates at each child’s own values.

Over 95% of the sample had complete ACEs data; 1% of respondents were missing data for all of the ACEs, and 2.3% of respondents were missing data for any ACE. For the resilience variable, the sample had 0.2% missing data. For these sets of missing data, they were excluded, as they were missing at random and less than 5% [32]. Also, the household income-to-poverty variable had 9% missing data in the NSCH. For this variable only, we used multiple imputations with five replications that were provided by the State and Local Area Integrated Telephone Survey and incorporated them into our analysis. All analyses were conducted with Stata (Version 13; Stata Corp, College Station, TX), to incorporate consideration of the complex survey sample. All analyses were adjusted with stratified sampling weights provided in the NSCH public use data set, to permit national inferences.

## Results

### Sample characteristics and individual child-level factors

In 2011–12 among 62,200 children 6–17 years old, nearly 68% of children were reported as having resilience and 32% of children were not. Less than one-half of children in the sample had no ACEs (47%); 26% had 1 ACE, 19% had 2–3 ACEs, 8% had 4 ACEs or more.

For children with 4 or more ACEs, the mean age was higher than children with no ACEs (*p* < .001; Table 1). The frequency of ACEs differed by race and ethnicity (*p* < .001; Table 1). In addition, a greater proportion of children with ACEs than without ACEs were children with special health care needs (p < .001; Table 1). Children with any number of ACEs were more likely to live under 200% of the federal poverty level (p < .001; Table 1) and to have parents with a high school education or less, when compared with children without ACEs (p < .001; Table 1). Children with any ACEs were less likely to live in a two-parent family, when compared with children with no ACEs (p < .001) (Additional file 1: Table S1; Table 1).

**Table 1:** Study Sample Characteristics by Adverse Childhood Experiences, NSCH 2011–2012†

	Sample	0 ACEs	1 ACE	2–3 ACEs	4+ ACEs	*P*-value
	(n = 62,200)	(*n* = 32,724) (47%)	(*n* = 14,907) (26%)	(*n* = 10,179) (19%)	(*n* = 4390) (8%)	
Weighted Proportion	No. (%)	No. (%)	No. (%)	No. (%)	No. (%)	
Age, mean (SD)	11.5 (3.5)	11.2 (3.7)	11.6 (3.4)	11.9 (3.2)	12.3 (3.2)	< .001
Gender, % male	32,142 (51.1)	16,850 (51.3)	7707 (51.1)	5267 (50.5)	2318 (52.6)	N.S.
Race/Ethnicity						< .001
Non-Hispanic White	41,915 (54.4)	23,851 (58.9)	9406 (51.0)	6129 (49.6)	2529 (50.9)	
Non-Hispanic Black	5733 (13.9)	2115 (9.9)	1778 (16.1)	1355 (19.2)	485 (16.9)	
Hispanic	7673 (22.2)	3449 (21.3)	2110 (24.5)	1432 (22.2)	682 (20.9)	
Other race/ethnicity	6260 (9.5)	2976 (9.9)	1449 (8.5)	1176 (9.08)	659 (11.4)	
Child w/special health care need	15,314 (24.0)	6404 (18.6)	3651 (23.6)	3289 (30.1)	1970 (42.8)	<.001
Household poverty status						< .001
0–133% FPL	12,829 (29.6)	3382 (18.2)	3743 (34.1)	3655 (41.9)	2049 (52.9)	
134–200% FPL	6192 (12.2)	2236 (9.59)	1844 (13.8)	1444 (14.8)	668 (15.6)	
201% FPL or greater	43,179 (58.2)	27,106 (72.2)	9320 (52.1)	5080 (43.3)	1673 (31.5)	
Parental education						< .001
Less than high school	3533 (11.5)	1186 (8.6)	1089 (14.0)	842 (13.5)	416 (15.4)	
High school graduate	9409 (19.8)	3353 (14.8)	2745 (22.3)	2235 (25.8)	1076 (26.5)	
Greater than high school	48,924 (68.7)	28,010 (76.6)	10,984 (63.7)	7046 (60.7)	2884 (58.1)	
Total children in household						
One child	24,863 (21.2)	11,953 (18.4)	6380 (22.6)	4614 (25.2)	1916 (24.2)	<.001
Two children	23,867 (38.4)	13,753 (41.8)	5315 (37.8)	3277 (34.2)	1342 (30.2)	
Three children	9382 (27.6)	5034 (28.7)	2167 (26.8)	1478 (25.1)	703 (29.7)	
Four or more children	4268 (12.8)	1984 (11.1)	1045 (12.8)	810 (15.5)	429 (15.8)	
Family structure						< .001
Two parent family	47,677 (73.5)	30,524 (91.3)	10,371 (68.9)	5203 (50.5)	1579 (38.1)	
Single mother	9812 (19.3)	1491 (6.7)	3138 (22.6)	3495 (36.2)	1688 (42.3)	
Other family type	4433 (7.2)	583 (2.0)	1314 (8.5)	1439 (13.3)	1097 (19.6)	
Attends religious service						
Not often	42,348 (70.6)	9370 (26.4)	4929 (31.2)	3641 (32.6)	1671 (33.4)	<.001
Often	19,611 (29.4)	23,244 (73.6)	9904 (68.8)	6496 (67.4)	2704 (66.6)	
Family eats together, mean days (SD)	5.0 (2.1)	5.1 (2.1)	5.0 (2.0)	4.9 (2.1)	5.0 (2.1)	**
Shares ideas with children						<.001
Not well	1639 (3.2)	543 (1.8)	413 (3.3)	407 (4.8)	276 (7.1)	
Well	60,524 (96.8)	32,162 (98.2)	14,485 (96.7)	9766 (95.2)	4111 (92.9)	
Neighborhood cohesion						< .001
Not cohesive	7301 (15.9)	2370 (10.3)	2004 (17.4)	1839 (21.9)	1088 (29.5)	
Cohesive	53,648 (84.1)	29,763 (89.7)	12,584 (82.6)	8082 (78.1)	3219 (70.5)	
Neighborhood safety						< .001
Unsafe	5482 (13.0)	1751 (8.5)	1612 (14.6)	1369 (18.3)	750 (21.1)	
Safe	56,442 (87.0)	30,834 (91.5)	13,222 (85.4)	8762 (81.7)	3624 (78.9)	
Neighborhood amenities						
0–1	6215 (9.9)	2961 (8.7)	1570 (10.6)	1123 (11.1)	561 (12.3)	<.001
2	7899 (12.2)	3936 (10.9)	1979 (13.2)	1372 (13.3)	612 (13.6)	
3	15,108 (24.3)	7857 (23.5)	3642 (23.6)	2521 (26.2)	1088 (26.6)	
4	32,159 (53.6)	17,577 (56.9)	7505 (52.6)	5003 (49.4)	2074 (47.5)	
Neighborhood detractors						<.001
None	45,639 (72.1)	25,955 (80.1)	10,633 (70.9)	6573 (62.4)	2448 (51.3)	
1	10,495 (17.1)	4780 (13.6)	2723 (18.7)	2027 (20.6)	965 (24.1)	
> 2	5797 (10.8)	1851 (6.2)	1454 (10.4)	1531 (16.9)	961 (24.6)	
Presence of mentors, yes	57,694 (89.0)	30,640 (89.3)	13,624 (87.1)	9376 (90.5)	4054 (90.1)	<.01

*Abbreviations*: *N.S.* Not Significant, *FPL* Federal Poverty Line; amenities include: Presence of sidewalks, parks, recreation centers, and libraries;

Detractors include: litter, rundown housing, and graffiti

†Numbers listed are unweighted; however all proportions are displayed as weighted %

*P*-values reflect statistical comparisons across the categories of ACEs

**Comparison are all to 0 ACEs; *p* = 0.06 for 1 ACE, p < .001 for 2–3 ACEs, and *p* = 0.01 for 4+ ACEs

### Relationship between ACEs and resilience

**Fig. 1:**
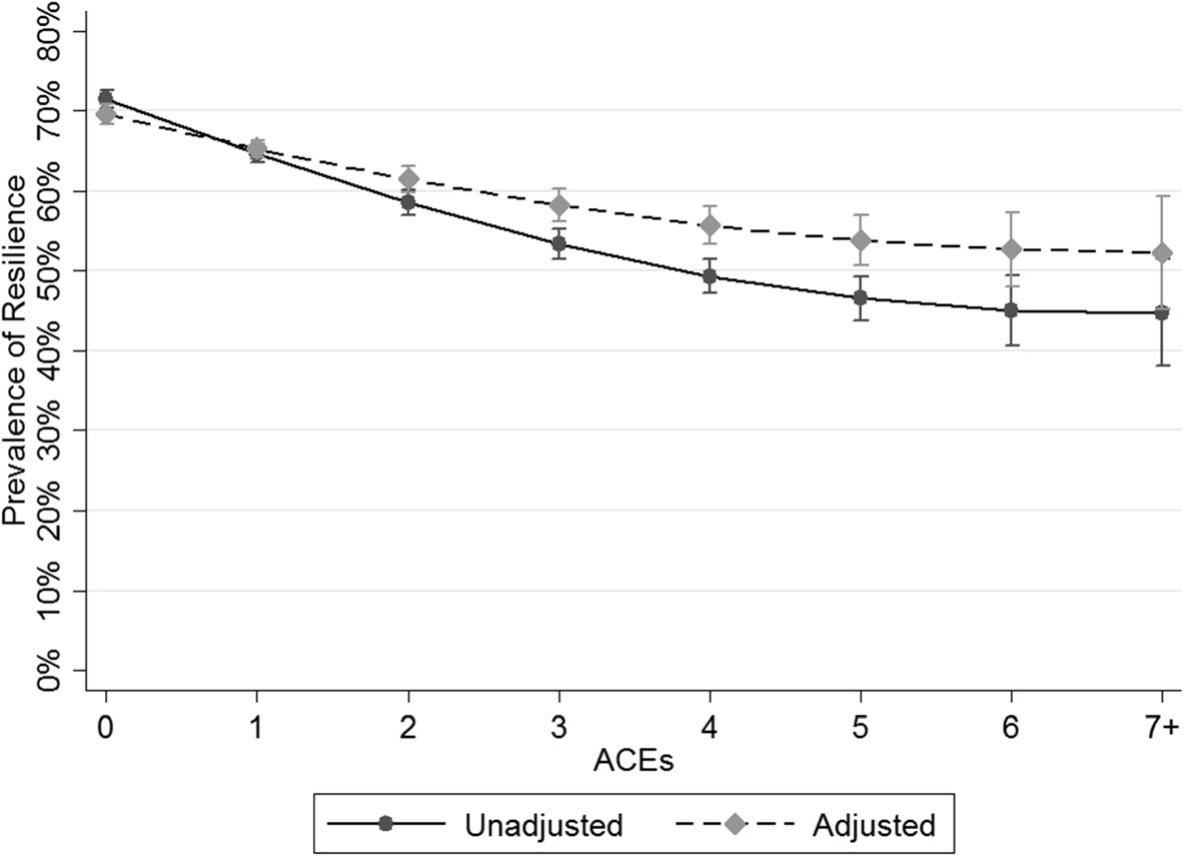
Unadjusted and Adjusted Probability of Resilience by Number of NSCH-Adverse Childhood Experiences (NSCH-ACEs)

As the ACE score increased, the probability of parent-perceived resilience decreased for children (Fig. 1). Children with 0 ACEs, had a 70% adjusted probability of resilience, compared with children with 1 ACE at 65%, children with 2 and 3 ACEs at 61 and 58%, respectively, and children with 4 or more ACEs with 56% adjusted probability of parent-reported resilience or less. While the stepwise decrease in reported resilience persisted with higher levels of ACEs, the incremental change diminished at higher ACE scores. Adjustments for child, family, and neighborhood-level factors attenuated the decrement in resilience associated with ACEs; however, the relationship still persisted (Fig. 1).

### Family-level factors and resilience

**Fig. 2:**
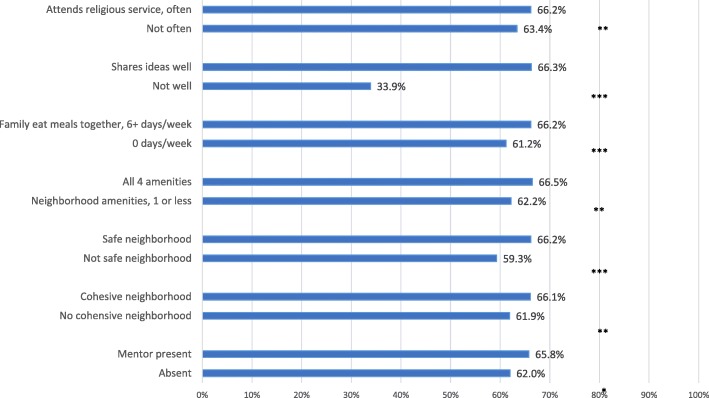
Adjusted Probability of Resilience by Family and Community-Level Factors†. † Adjusted for Child factors (e.g., child’s age, race/ethnicity, sex, special health care needs status, ace score); Family factors (e.g., (household income-to-poverty ratio, parental education, number of siblings, family structure, eating meals together, sharing ideas together); and Community factors (e.g., neighborhood cohesion, safety, amenities, such as the presence of sidewalks, parks, recreation centers, or libraries), and detractors, such as litter, rundown housing, graffiti; and mentorship. *p < .05 **p < .01 ***p < .001

When examining family-level characteristics, children in families that ate meals together six days per week had a higher probability of parent-perceived resilience compared with children whose families did not eat meals together at all (*p* < .001; Fig. 2). Furthermore, children in families that attended religious services together were more likely to be described as resilient compared to children in families who did not participate in these activities (*p* < 0.01; Fig. 2). And, children in families that shared ideas had a higher probability of resilience than those children whose families did not (p < .001; Fig. 2).

### Community-level factors and resilience

Children in neighborhoods that parents considered safe (p < .001; Fig. 2) and cohesive (*p* < .01; Fig. 2) were more likely to be perceived as having resilience by their parents. Children in neighborhoods with all 4 amenities (i.e., sidewalks, recreation centers, libraries, and parks) were more likely to demonstrate resilience than children in neighborhoods with 1 amenity or less (p < .01; Fig. 2). Finally, the presence of a mentor for a child was independently, positively associated with resilience (*p* < .05; Fig. 2).

## Discussion

Our findings illustrate a dose-response relationship between NSCH-ACEs and a child’s parent-perceived resilience, as measured by self-regulation—the greater the number of ACEs, the lower the probability of resilience, even after controlling for a number of child, family, and neighborhood factors. We also identify potentially modifiable family and community factors independently associated with resilience, such as families sharing ideas together and living in a neighborhood with multiple amenities. While many studies focus on ACEs and long-term health in adults, few studies have linked ACEs and parent perceptions of resilience in childhood. Resilience is an important factor to investigate, as it has been examined as a protective factor in the development of both anti-social behavior [23] and post-traumatic stress disorder (PTSD) [33–35] and is also an important factor in the relationship between emotional neglect and psychiatric symptoms [36, 37]. Our study aligns with existing literature and further elucidates the relationship of ACEs with resilience development and key resilience-promoting community and family-level factors [3, 37]. This study extends knowledge about ACEs by examining a positive outcome, such as resilience. Focusing on resilience in children may serve as important starting place for the development of effective interventions in childhood to mitigate ACEs.

The negative dose-response relationship between the number of ACEs and probability of resilience is evident. While the stepwise decline in resilience seems to be most pronounced for children with one to three ACEs, resilience is lower with each additional ACE even at higher ACE scores. Nonetheless, our findings support prior research demonstrating that many individuals exposed to adversity still demonstrate resilience [38]. Our work explores the relationship between ACEs and resilience in more depth. We also highlight the family factors (e.g., sharing ideas, attending religious services, eating meals together) and community amenities (e.g., sidewalks, recreation centers, libraries, and parks) that may protect or promote resilience in children with and without ACEs.

Also, certain groups of children disproportionately experience ACEs, which may intensify the need to understand both the impact of cumulative adversities on children and the protective and promoting factors of resilience. Demographically, these groups include non-Hispanic black children, children of lower socioeconomic status, and children with special health care needs. ACEs can be particularly stressful adversities for children, because many directly impact the family and the family is meant to be a child’s first barrier against adversity. The implications of a link between higher ACE score and resilience are myriad. Screening for resilience could help healthcare providers identify and stratify children at greatest risk for poor health outcomes. For example, children with a high ACE score and low levels of resilience, may be identified more readily and benefit from more intense support. Additionally, as the emphasis on prevention, screening, and treatment of ACEs continues to grow, it will be important to understand the role of resilience in mitigating poor health outcomes for individuals with ACEs and how factors promoting resilience might be a future area for intervention.

While many studies examine individual characteristics that promote resilience, [35, 39] some of the most important factors that protect and promote resilience appear to be external to the individual, such as caregiver and family support and cultural and community environments. Our findings reinforce that family factors, such as sharing meals and attending religious services together, are independently associated with resilience [21, 39]. Additionally, we found children in families that share ideas together are more likely to demonstrate resilience. Enhanced interactions may improve self-regulatory behaviors and increase parental insights about their child’s ability to self-regulate. This relationship has been previously demonstrated in children with emotional, mental, or behavioral problems [3]. Children with emotional, mental, or behavioral problems that are in families that exchange ideas and discuss topics of significance have higher reported resilience [3]. These family factors might be mechanisms that foster resilience in children with and without ACE exposure. Additionally, these factors may guide the clinician and child welfare professional’s recommendations for parents, guardians, and extended family members to promote child resilience.

Potentially modifiable community-level factors may also contribute to resilience in children [39, 40]. Our findings support other research showing that mentorship,[40] neighborhood safety, and neighborhood cohesion, which may serve as markers of resourced neighborhoods, were associated with resilience in the general population of children [17, 39]. Additionally, we found that children have a higher likelihood of resilience when living in communities with certain amenities. Particularly, having all four neighborhood amenities of interest in this survey were associated with resilience in children, compared with children living in neighborhoods with only one type of amenity or no reported amenities. Intuitively, neighborhoods that are safe, supportive, and offer recreational opportunities are better for children. Our study highlights some specific aspects of neighborhoods that may be associated with child resilience and might represent opportunities for local policymakers to prioritize community assets. Furthermore, mentoring was independently associated with resilience and points to the role that trusted, supportive adults outside the household might play in promoting child resilience [20, 24, 41].

Some health care organizations, such as medical clinics and hospitals, have already begun to address ACEs as part of clinical care. These settings have begun to actively screen for ACEs, provide education to families about ACEs, or collaborate with non-traditional partners [42]. Others have begun to implement trauma-informed care approaches in practice, as supported by the American Academy of Pediatrics, the Substance Abuse and Mental Health Administration (SAMHSA) and the Centers for Medicare & Medicaid Services (CMS) [43, 44]. Interdisciplinary collaborations among health care, social services, the justice system, policymakers, and community partners can help to foster resilience in ACE-exposed children. For example, providers could recommend or collaborate with local mentoring organizations, after-school or recreation programs, and early childhood education programs for patients at-risk. Additionally, established partnerships with key stakeholders, like policymakers, may allow community leaders to advocate for resources, such as recreation centers, libraries, and parks, which may enhance community resources, bolstering resilience for children in those neighborhoods.

### Limitations

The findings should be interpreted in light of the study limitations. The cross-sectional survey design precludes us from firm conclusions about a causal relationship between ACEs and parent-perceived resilience. The data indicates a dose-response relationship, while suggestive of a causal pathway, still requires further inquiry. Additionally, the exposure and outcome measures themselves have limitations. For example, the ACE score does not capture information regarding the frequency, duration, and severity of the adversities that children experienced, and does not include all the adversities a child might experience, such as bullying and poor peer relationships. However, this is also a limitation of previous ACE studies. The ACE score also assumes an equivalency in the impact of different specific adversities, which may not be truly equivalent for specific children or across the population. Further, the ACEs collected in this dataset (NSCH-ACEs) are parent-reported, modified from the original ACEs, and do not include the categories of abuse and neglect. This data may not have been collected due to concerns of refusal to answer due to fear of investigation or prosecution. Also, the data relied on parent-report of ACEs, the actual ACE numbers could have been underreported, as the parents, themselves could have directly contributed to their children having ACEs. While the NSCH used modified ACE measures, Bethell et al., published a recent study that examined the validity of the modified ACE measures and found that the NSCH-ACEs could be risk scored cumulatively and demonstrated predictive validity [45].

Another important limitation is the definition of resilience itself. In this study, resilience was defined as staying calm and in control when faced with a challenge, which represents a parent’s perception of the self-regulation aspect of resilience but may not encompass other aspects of resilience, such as optimism or intellect. However, this definition has been used in other child-focused ACEs studies [3, 9]. Additionally, there is little agreement on the definition, measurement, and application of resilience in research [46]. For this study, the challenges were defined as ACEs; however, children with 0 ACEs were still perceived as having resilience. While the authors defined ACEs as significant challenges, there may have been additional challenges that were not captured by the ACEs used in this study, which may account for children being described as resilient in the absence of ACEs.

## Conclusion

Professionals in a variety of settings, such as schools, clinics, and daycares, are increasingly expected to identify children with ACEs and intervene in order to ameliorate both the adversity and its impact and to improve child outcomes. One area for potential study and intervention is identifying resilience as a buffer of the poor outcomes associated with ACEs and also if the resilience-promoting factors differ for children with ACEs compared to children without ACEs. Further, if resilience mitigates the impact of ACEs, child professionals may need to understand key ways to promote resilience. Since resilience is a dynamic process that can be modulated [47–50] equipping communities, families, and providers with a better understanding of resilience and its supporting factors is an important step towards strengthening and protecting families. Future work could be aimed at determining which ACEs have a more detrimental impact on resilience. Additionally, research that investigates the use of resilience screening in primary health care settings as well as identifies key family and community factors that best protect and promote resilience in ACE-affected children is needed. This work could enable targeted interventions and judicious use of community resources. Emphasis on the social ecology of the child (e.g. the nuclear family, extended family and neighbors, neighborhood and community, culture, policy) makes potential interventions more easily identifiable and multi-faceted. Our society bears a responsibility to protect children from experiencing ACEs, increasing prevention as well as protective factors, so that every child can flourish and reach their full potential. Children cannot make themselves resilient—resilience is nurtured through relationships and exposures to experiences and resources that promote it. Many service providers can play a role in facilitating children’s resilience through the guidance they offer in their offices, linkages with community resources, and advocacy for policies and resources that promote resilience.

## Additional file

Additional file 1: **Table S1.** Questions from the National Survey of Children’s Health 2011–2012†. †Please see 2012 NSCH: Child Health Indicator and Subgroups SPSS Codebook, Version 1.0 for more information on coding used regarding these questions. Questions from the National Survey of Children’s Health used in the study. (DOCX 20 kb)

## Abbreviations

ACEs: Adverse Childhood Experiences
CMS: Centers for Medicare & Medicaid Services
FPL: federal poverty level
NSCH: National Survey of Children’s Health
NSCH-ACEs: National Survey of Children’s Health Adverse Child Experiences
PTSD: post-traumatic stress disorder
SAMHSA: Substance Abuse and Mental Health Administration

## References

[1] Felitti VJ, Anda RF, Nordenberg D (1998). Relationship of childhood abuse and household dysfunction to many of the leading causes of death in adults. The Adverse Childhood Experiences (ACE) Study. Am J Prev Med.

[2] Anda RF, Croft JB, Felitti VJ (1999). Adverse childhood experiences and smoking during adolescence and adulthood. JAMA.

[3] Bethell C, Gombojav N, Solloway M, Wissow L (2016). Adverse childhood experiences, resilience and mindfulness-based approaches: Common Denominator Issues for Children with Emotional, Mental, or Behavioral Problems. Child Adolesc Psychiatr Clin N Am.

[4] Schilling EA, Aseltine RH, Gore S (2007). Adverse childhood experiences and mental health in young adults: a longitudinal survey. BMC Public Health.

[5] Chapman DP, Whitfield CL, Felitti VJ, Dube SR, Edwards VJ, Anda RF (2004). Adverse childhood experiences and the risk of depressive disorders in adulthood. J Affect Disord.

[6] Dube SR, Anda RF, Felitti VJ, Edwards VJ, Croft JB (2002). Adverse childhood experiences and personal alcohol abuse as an adult. Addict Behav.

[7] CDC. About the CDC-Kaiser ACE Study. CDC-Kaiser ACE Study. https://www.cdc.gov/violenceprevention/acestudy/about.html. Published June 14, 2016. Accessed November 21, 2016.

[8] Jimenez M, Wade R, Lin Y, Morrow L, Reichman N (2016). Adverse experiences in early childhood and kindergarten outcomes. Pediatrics.

[9] Bethell CD, Newacheck P, Hawes E, Halfon N (2014). Adverse childhood experiences: assessing the impact on health and school engagement and the mitigating role of resilience. Health Aff Proj Hope.

[10] Hart H, Rubia K (2012). Neuroimaging of child abuse: a critical review. Front Hum Neurosci.

[11] Cohen RA, Grieve S, Hoth KF (2006). Early life stress and morphometry of the adult anterior cingulate cortex and caudate nuclei. Biol Psychiatry.

[12] Brockie TN, Heinzelmann M, Gill J. A framework to examine the role of epigenetics in health disparities among native Americans. Nurs Res Pract. 2013;2013 10.1155/2013/410395. 10.1155/2013/410395 PMC3872279 24386563

[13] Kim-Cohen J, Moffitt TE, Caspi A, Taylor A (2004). Genetic and environmental processes in young children’s resilience and vulnerability to socioeconomic deprivation. Child Dev.

[14] Bellis MA, Hardcastle K, Ford K (2017). Does continuous trusted adult support in childhood impart life-course resilience against adverse childhood experiences - a retrospective study on adult health-harming behaviours and mental well-being. BMC Psychiatry.

[15] Yehuda R, Flory JD (2007). Differentiating biological correlates of risk, PTSD, and resilience following trauma exposure. J Trauma Stress.

[16] Masten AS (2015). Ordinary Magic - Resilience in Development.

[17] Masten AS (2004). Regulatory processes, risk, and resilience in adolescent development. Ann N Y Acad Sci.

[18] Masten Ann S (2014). Global perspectives on resilience in children and youth. Child Dev.

[19] Garmezy N. Stress-resistant children: The search for protective factors. In: Stevenson JE, editor. Recent Research in Developmental Psychopathology: Journal of Child Psychology and Psychiatry Book Supplement #4. Oxford: Pergamon Press; 1985. p. 213–33.

[20] Masten AS (2011). Resilience in children threatened by extreme adversity: frameworks for research, practice, and translational synergy. Dev Psychopathol.

[21] Kasen S, Wickramaratne P, Gameroff MJ, Weissman MM (2012). Religiosity and resilience in persons at high risk for major depression. Psychol Med.

[22] Sege R, Linkenbach J (2014). Essentials for childhood: promoting healthy outcomes from positive experiences. Pediatrics.

[23] Gardner TW, Dishion TJ, Connell AM (2008). Adolescent self-regulation as resilience: resistance to antisocial behavior within the deviant peer context. J Abnorm Child Psychol.

[24] Luthar SS, Cicchetti D, Cohen DJ (2006). Resilience in development: a synthesis of research across five decades. Developmental psychopathology: Risk, Disorder, and Adaptation.

[25] Rothbart MK, Posner MI (2005). Genes and experience in the development of executive attention and effortful control. New Dir Child Adolesc Dev.

[26] Artuch-Garde R, M del C G-T, de la Fuente J, Vera MM, Fernández-Cabezas M, López-García M. Relationship between resilience and self-regulation: a study of Spanish youth at risk of social exclusion. Front Psychol. 2017;8 10.3389/fpsyg.2017.00612. 10.3389/fpsyg.2017.00612 PMC5397523 28473792

[27] Bronfenbrenner U. The Ecology of Human Development: Experiments by Nature and Design. unknown edition. Cambridge, mass: Harvard University Press; 1s981.

[28] 2011–2012 National Survey of Children’s Health Frequently Asked Questions. April 2013. Available from URL: http://www.cdc.gov/nchs/slaits/nsch.htm.

[29] Bramlett MD, Blumberg SJ, Zablotsky B, et al. Design and Operation of the National Survey of Children’s Health, 2011–2012. Vital Health Stat Ser 1 Programs Collect Proced. 2017;59:1–256. 28796596

[30] Data Resource Center for Child and Adolescent Health, sponsored by the Maternal and Child Health Bureau. 2011/12 National Survey of Children’s Health. Child and Adolescent Health Measurement Initiative (CAHMI). http://childhealthdata.org/docs/drc/2011-12-nsch-sampling-and-administration.pdf?sfvrsn=1. Accessed December 7, 2017.

[31] 2011–2012 National Survey of Children’s Health SPSS Code for Data Users: Child Health Indicators and Subgroups. Codebook Version 1.0. http://childhealthdata.org/docs/nsch-docs/spsscodebook_-2011_2012_nsch_v1_all.pdf. Published April 2013. Accessed December 7, 2017.

[32] Dong Y, C-YJ P. Principled missing data methods for researchers. Springerplus. 2013;2 10.1186/2193-1801-2-222. 10.1186/2193-1801-2-222 PMC3701793 23853744

[33] Bensimon M (2012). Elaboration on the association between trauma, PTSD and posttraumatic growth: the role of trait resilience. Personal Individ Differ.

[34] Lepore S, Revenson T (2006). Resilience and posttraumatic growth: recovery, resistance, & reconfiguration. Handbook of posttraumatic growth: research and practice.

[35] Bonanno GA (2004). Loss, trauma, and human resilience: have we underestimated the human capacity to thrive after extremely aversive events?. Am Psychol.

[36] Campbell-Sills L, Cohan SL, Stein MB (2006). Relationship of resilience to personality, coping, and psychiatric symptoms in young adults. Behav Res Ther.

[37] Hughes K, Ford K, Davies AR, Homolova L, Bellis MA (2018). Sources of resilience and their moderating relationships with harms from adverse childhood experiences.

[38] Tummala-Narra P (2007). Conceptualizing trauma and resilience across diverse contexts. J Aggress Maltreatment Trauma.

[39] Jaffee SR, Caspi A, Moffitt TE, Polo-Tomás M, Taylor A (2007). Individual, family, and neighborhood factors distinguish resilient from non-resilient maltreated children: a cumulative stressors model. Child Abuse Negl.

[40] Southwick SM, Morgan CA, Vythilingam M, Charney D (2007). Mentors enhance resilience in at-risk children and adolescents. Psychoanal Inq.

[41] Burt KB, Douglas Coatsworth J, Masten AS. Competence and psychopathology in development. In: Cicchetti D, editor. Developmental Psychopathology: Wiley; 2016. 10.1002/9781119125556.devpsy409.

[42] Center for Youth Wellness Clinical Programs. http://www.centerforyouthwellness.org/what-we-are-doing/clinical-programs. Accessed March 28, 2017.

[43] Dowd D, Forkey H, Gillespie R, Pettersen T, Spector L, Stirling J. Addressing adverse childhood experiences and other types of trauma in the primary care setting. Trauma Toolbox for Primary Care. 2014. https://www.aap.org/en-us/Documents/ttb_addressing_aces.pdf. Accessed 7 Dec 2017.

[44] SAMHSA. Clinical Practice and Trauma Informed Care. https://www.integration.samhsa.gov/integrated-care-models. Accessed 9 Sept 2017.

[45] Bethell CD, Carle A, Hudziak J (2017). Methods to assess adverse childhood experiences of children and families: toward approaches to promote child well-being in policy and practice. Acad Pediatr.

[46] Aburn G, Gott M, Hoare K (2016). What is resilience? An integrative review of the empirical literature. J Adv Nurs.

[47] Fergus S, Zimmerman MA (2005). Adolescent resilience: a framework for understanding healthy development in the face of risk. Annu Rev Public Health.

[48] Luthar SS. Resilience and Vulnerability: adaptation in the context of childhood adversities: Cambridge University Press; 2003.

[49] Egeland B, Carlson E, Sroufe LA (1993). Resilience as process. Dev Psychopathol.

[50] Luthar SS, Cicchetti D, Becker B (2000). The construct of resilience: a critical evaluation and guidelines for future work. Child Dev.

