# Supplementary material for: Childhood adversity and parent perceptions of child resilience
Source: BMC Pediatr. 2018 Jun 26;18:204. doi: 10.1186/s12887-018-1170-3 (PMC6020317; doi:10.1186/s12887-018-1170-3)
Supplement: Supplementary file 1 — Table S1. Questions from the National Survey of Children’s Health 2011–2012†. †Please see 2012 NSCH: Child Health Indicator and Subgroups SPSS Codebook, Version 1.0 for more information on coding used regarding these questions. Questions from the National Survey of Children’s Health used in the study. (DOCX 20 kb) [file 12887_2018_1170_MOESM1_ESM.docx]

**Table S1. Questions from the National Survey of Children’s Health 2011-2012†**

| **Resilience (Main dependent variable)** |
| --- |
| [He/She] stays calm and in control when faced with a challenge |
| **NSCH-Adverse Childhood Experiences (Main exposure variable)** |
| Since child was born, how often has it been very hard to get by on your family's income, for example, it was hard to cover the basics like food or housing? |
| Did child ever live with a parent or guardian who got divorced or separated after [S.C.] was born? |
| Did child ever live with a parent or guardian who died? |
| Did child ever live with a parent or guardian who served time in jail or prison after [S.C.] was born? |
| Did child ever see or hear any parents, guardians, or any other adults in [his/her] home slap, hit, kick, punch, or beat each other up? |
| Was [S.C.] ever the victim of violence or witnessed any violence in [his/her] neighborhood? |
| Did child ever live with anyone who was mentally ill or suicidal, or severely depressed for more than a couple of weeks? |
| Did child ever live with anyone who had a problem with alcohol or drugs? |
| Was child ever treated or judged unfairly because of [his/her] race or ethnic group? |
| **Covariates** |
| ***Child-Level Factors*** |
| Selected child's age in years at interview |
| Sex of selected child |
| Race classification of child (White, Black, Other) and Ethnicity of child |
| Child with special health care need |
| Poverty level of this household based on US Department Health and Human Services poverty guidelines |
| Highest education attained among parents or guardian |
| How many people less than 18 years old live in this household |
| Combined family structure and marital/cohabitation status of child's parent(s) in the household |
| ***Family-Level Factors*** |
| About how often does child attend a religious service? |
| How well can you and child share ideas or talk about things that really matter? |
| During the past week, on how many days did all the family members who live in the household eat a meal together? |
| ***Community-Level factors*** |
| How often do you feel child is safe in your community or neighborhood? |
| *Neighborhood cohesion:*  People in this neighborhood help each other out.  We watch out for each other's children in this neighborhood.  There are people I can count on in this neighborhood.  If my child were outside playing and got hurt or scared, there are adults nearby who I trust to help my child. |
| *Neighborhood amenities:*  Do sidewalks or walking paths exist in your neighborhood?  Does a park or playground area exist in your neighborhood?  Does a recreation center, community center, or boys' or girls' club exist in your community?  Does a library or bookmobile exist in your community? |
| *Neighborhood detractors:*  In your neighborhood, is there litter or garbage on the street or sidewalk?  How about poorly kept or [dilapidated/rundown] housing?  How about vandalism such as broken windows or graffiti? |
| Other than adults in your home or child's parents, is there at least one other adult in the child’s school, neighborhood, or community who knows [him/her] well and who [he/she] can rely on for advice or guidance? |

†Please see 2012 NSCH: Child Health Indicator and Subgroups SPSS Codebook, Version 1.0 for more information on coding used regarding these questions.
